# A Rasch Analysis of the Irrational Procrastination Scale (IPS)

**DOI:** 10.3389/fpsyg.2020.615341

**Published:** 2021-01-12

**Authors:** Amy Shaw, Jennifer J. Zhang

**Affiliations:** ^1^Department of Psychology, Faculty of Social Sciences, University of Macau, Taipa, Macau; ^2^Department of Psychology, University of Houston, Houston, TX, United States

**Keywords:** procrastination, irrational procrastination scale, Rasch analysis, rating scale model, dimensionality, measurement

## Abstract

The present study analyses the psychometric properties of the irrational procrastination scale (IPS; Steel, 2002, 2010) in a sample of United States college students using the Rasch modeling approach. Results showed that the IPS items had a high level of reliability, good content validity, structural validity, and substantive validity, and no differential item functioning (DIF) effects in terms of gender. The IPS was found to be unidimensional, supporting the originally proposed theoretical structure by Steel (2002, 2010). Finally, psychometric implications derived from the results and study limitations are discussed; recommendations for future investigations are also offered.

## Introduction

Defined as the inclination “to voluntarily delay an intended course of action despite expecting to be worse off for the delay” (Steel, 2007, p. 66), procrastination is predominantly viewed as an irrational/dysfunctional delay resulting from the failure of self-regulation or self-control (Steel, 2007, 2010) and often leading to lower task performance and decreased subjective well-being (Steel, 2007; Klingsieck, 2013). Past epidemiological research has established that procrastination is a phenomenon seemingly ubiquitous in both general public and academic settings: 20–30% of the general adult population regarded themselves as having procrastination issues (Harriott and Ferrari, 1996) whereas at least 50% of the student population reported having recurrent difficulties in fulfilling their academic commitments (Day et al., 2000; Steel and Ferrari, 2013).

The rise in recognition of the prevalence and negative consequences of procrastination has inspired growing efforts to validate and refine instruments for this construct (see Steel, 2010, for an overview). Despite the existence of various procrastination scales derived from different theoretical frameworks in the literature, the meta-analysis conducted by Steel (2010) found little empirical support for the assumption about dividing procrastination into multiple distinct subtypes; instead, Steel (2010) concluded that procrastination is best conceived of as a single unitary construct – the irrational or dysfunctional delay of actions in the implemental phase despite expecting it to be disadvantageous, which is considered as the core component of procrastination (Steel, 2007). Explicitly consistent with this conceptualization, the irrational procrastination scale (IPS; Steel, 2002, 2010) was devised to assess the irrational delay tendency. The IPS consists of nine items [e.g., “My life would be better if I did some activities or tasks earlier” (Item 3)] and three items are reversely scored (Items 2, 6, and 9). Since its inception, the IPS has attracted considerable attention from the research community, primarily due to its convenience to use and the simplicity of the unidimensional theoretical structure (Steel and Ferrari, 2013). Empirical evidence from past work has largely pointed to the unidimensionality of the IPS (e.g., Svartdal et al., 2016; Svartdal, 2017; Kim et al., 2020) – although two studies (Prayitno et al., 2013; Rozental et al., 2014) found the reversely scored items to load on a different factor compared to the rest of the items on the scale, these authors concluded that this could just be a reflection of a methodological artifact (Schmitt and Stuits, 1985). There is also converging evidence from several studies demonstrating the psychometric soundness of the IPS regarding internal consistency and relations to other instruments (Rebetez et al., 2014; Rozental et al., 2014; Svartdal and Steel, 2017; Guilera et al., 2018).

Notably, previous studies on the construct validity and other psychometric properties of the IPS were mainly conducted in the lens of Classical Test Theory (CTT). A major weakness of CTT-based approaches to examining the dimensionality of scales is that these methods presume the existence of linear relationships between the variables and factors – a condition that numerous measures (including the IPS) could not satisfy (Hambleton et al., 2000). Also, because CTT methods are suitable for linear and interval data, it is problematic to apply such methods directly to non-interval raw data derived from Likert rating scales without appropriate data reconstruction (Embretson and Reise, 2000; Hambleton et al., 2000). Hence, it is imperative to apply alternative psychometric methods that may compensate the limitations inherent to the assumptions and procedures of CTT. This could involve the use of modern item response theory (IRT), for example, Rasch analysis, in order to improve the precision and effectiveness of the analyses (Wright and Masters, 1982; Wright, 1997; Embretson and Reise, 2000; Hambleton et al., 2000). Furthermore, Rasch analysis explores item and person fit and provides valuable item-level information (e.g., people and item parameters) that could be useful for scale refinement and calibration (Wright and Linacre, 1989). As such, Rasch analysis not only can be used as a confirmatory test of the unidimensionality, but also produces detailed diagnostic information about the quality of the measurement that complements CTT results [Andrich, 2011; for a thorough overview of the advantages of Rasch models over CTT-based methods, see Bond and Fox (2007) and Wright and Masters (1982); also see Balsamo et al. (2019) for a recent example of applying Rasch analysis to overcome CTT drawbacks in the development and refinement of measures]. Additionally, given some empirical evidence (albeit the effect sizes are small and weak) concerning gender differences in procrastination (men tended to procrastinate more than women; Steel and Ferrari, 2013), Rasch analysis is useful in determining whether the IPS items work similarly for male and female groups with differential item functioning (DIF), which would help determine whether possible gender differences in the scale scores are attributable to underlying trait differences.

Therefore, the purpose of the present study is to examine the psychometric properties of the IPS within the framework of Rasch modeling. Specifically, reliability, content validity, structural validity, substantive validity, and gender-based DIF of the scale are evaluated. To our knowledge, there have been no attempts at applying Rasch analysis for the IPS.

## Materials and Methods

### Procedure and Participants

The sample is comprised of *N* = 382 college students who participated in the study through SONA system for research credit rewards at a large public university in the Southern United States. After providing their written informed consent, participants completed a standard demographic survey in addition to the nine-item IPS (Steel, 2002, 2010). The IPS was rated on a 5-point Likert scale (1 = Very seldom or not true of me; 5 = Very often true or true of me) with higher scores indicating higher levels of irrational procrastination. Participants’ ages ranged from 18 to 23 years old with a mean of 19.50 (*SD* = 0.75). The sample is slightly predominated by female students (*N* = 205; 53.7%). Self-reported ethnic information suggested that the sample was ethnically diverse (33.8% were Hispanic/Latino, 28.5% were Caucasian/White, 18.6% were African–American, 15.2% were Asian, and 3.9% selected Other for Ethnicity). In terms of college major, the majority of the sample was in Psychology (62.8%). All participants were included in the final analysis sample (*N* = 382).

### Analytical Strategy

Given the polytomous item format of the IPS (a 5-point Likert rating scale), the Rating Scale Model (RSM; Andrich, 1978) was adopted for parameter estimation based on Linacre’s (2002) recommendation. As an extension of the Rasch model for polytomous items, the RSM properly transforms raw rating-scale ordinal data relative to people’s responses on an interval scale (Wright and Mok, 2004) and offers sufficient and specific item and person fit statistics for evaluation in Likert scales without requiring a large sample size (Linacre, 2002; Bond and Fox, 2007). According to Andrich (1978), the RSM is provided by the formula:

$$L\,o\,g\,\left(P_{n\,i\,k}/P_{n\,i\,\left(k-1\right)}\right)=B_{n}-D_{i}-F_{k}$$

where *P*_*nik*_ represents the probability that person *n* would be observed in category *k* of item *i, P_*ni(k*_*_–_*_1__)_* represents the probability that person *n* would be in category *k*−*1* of item *i, B_*n*_* represents the latent ability level (i.e., level of irrational procrastination) of person *n*, *D*_*i*_ represents the difficulty of item *i* (i.e., difficulty for a respondent to endorse the item), and *F*_*k*_ represents the probability of being observed in category *k* relative to category *k*−*1.* This step calibration parameter *F*_*k*_ is thus a rating scale threshold defined as the location associated with the equal probability of observing the two adjacent categories *k*−*1* and *k*.

Therefore, the RSM allows non-linear raw rating-scale data to be converted into calibrated item and person measures on a common, linear interval-level scale (using the log-odds unit or logit; Andrich, 1978; Wright and Masters, 1982). The produced metrics are sample- and item-distribution free or independent in that the item difficulty and person ability can be separated from each other – this feature makes it possible to estimate item difficulty independent from the distribution of people comprising the sample as well as to estimate person ability level of the latent trait free of the distribution of individual items (Andrich, 1978; Schumaker, 2004). For more details about Rasch modeling and specifically the RSM, readers are encouraged to read excellent texts such as Bond and Fox (2007).

## Results

The Rasch analyses were conducted using the most widely used Rasch computer program Winsteps (Linacre, 2011). Several fit indices and item and person parameters were generated from the analyses for the evaluation of the psychometric properties of the IPS.

### Reliability

As presented in Table 1, the point-measure correlations (*r_*pm*_;* comparable with item-total correlations in CTT) of the nine items on the scale range from 0.58 to 0.74, indicating the absence of non-modeled dependence among the items and potentially a common underlying construct of all items (Linacre, 2011). In accordance with previous studies (Prayitno et al., 2013; Rozental et al., 2014; Svartdal and Steel, 2017; Guilera et al., 2018), the reversed items (Items 2, 6, 9) showed the lowest point-measure correlations, possibly reflecting a statistical artifact in reverse scoring (Schmitt and Stuits, 1985). Moreover, the values for the standard error (*SE*) in Table 1 range from 0.09 to 0.10 only, demonstrating a high level of measurement precision. Also, the values for the item separation reliability (a reliability estimator similar to Cronbach’s alpha in CTT) and the person separation reliability (a reliability estimator that measures the proportion of people variance not explained by measurement error) are 0.95 and 0.87, respectively, suggesting a high degree of reliability of the scale and a high level of estimation precision for most people (Bond and Fox, 2007; Andrich, 2011).

**TABLE 1:** The IPS item-level psychometric properties and principal component analysis (PCA) results.

Item	MNSQ		*D*_*i*_	*SE*	*r*_*pm*_	*SC*
	Infit	Outfit				
1	0.88	0.87	0.86	0.09	0.73	0.31
2	0.89	0.89	–0.35	0.09	0.58	–0.11
3	0.83	0.82	–0.13	0.10	0.71	0.37
4	1.43	1.42	0.03	0.10	0.71	0.12
5	0.96	0.95	–0.03	0.10	0.65	–0.19
6	0.83	0.81	0.08	0.10	0.64	–0.06
7	1.48	1.49	0.51	0.10	0.73	0.33
8	0.84	0.83	–0.78	0.10	0.74	–0.21
9	0.86	0.87	–0.15	0.10	0.65	–0.10
Mean	1.00	1.00	0.00	0.10	−	−
*SD*	0.26	0.27	0.47	0.00	−	−

**D*_*i*_, item location; *SE*, standard error; *r*_*pm*_, point-measure correlation; *SC*, structural coefficients from the standardized residual PCA; Infit, information-weighted mean square statistic; Outfit, outlier-sensitive mean square statistic; MNSQ, mean square fit indices.*

### Content Validity

The content validity of the IPS was primarily evaluated by the items’ mean square fit statistics (infit and outfit). Infit and outfit indices express the correspondence between observed and expected model parameters; appropriate fit values could indicate that the expected parameters represent the observed responses adequately. As shown in Table 1, the mean values for the infit and outfit statistics are both equal to the expected value of 1.00, indicating a perfect fit of the items to the overall scale (Wolfe and Smith, 2007). All but two of the items had the individual item fit values (both infit and outfit) within the acceptable interval of 0.60–1.40 as recommended by Wright and Linacre (1994); the two items exceeding this range had fit values between 1.42 and 1.49 – still less than the threshold value of 2.0 that may suggest potential distortion effects on the measurement system (Linacre, 2011). The items were thus homogeneous in terms of the content, contributed well to the overall scale, and no item appeared to be redundant or unproductive on the scale (Wright and Linacre, 1994). These results also provided initial evidence for the unidimensionality of the assessment data in that all items seemed to be defining a central construct for the scale.

### Structural Validity

To examine the internal structure (or structural validity in the Rasch context) of the IPS directly, a principal component analysis (PCA) of the standardized residuals was performed with controlling for the primary dimension in the Rasch model to determine the possibility of local dependency or a redundant secondary dimension not intended for the scale (Wolfe and Smith, 2007). As suggested by Linacre (2011), an eigenvalue below 3.00 in combination with a less than 10% of the unexplained variance as suggested by the first residual component in PCA could indicate unidimensionality. As presented in Table 1, the SCs (structural coefficients or standardized residual loadings in PCA) of all nine items are within the acceptable interval of −0.40 to 0.40, suggesting that all items on the scale corresponded to the defined construct well (Smith, 2004; Wolfe and Smith, 2007). The eigenvalue of the first residual component is 2.60 (below the cut-off point of 3.00) and represents a residual or unexplained variance of 9.4% (below the 10% threshold). These values did not exceed the criteria recommended by Linacre (2011) and thus suggested that the standardized residuals have no additional systematic information that might be indicative of a secondary dimension of the IPS. Furthermore, the variance explained by the total scale (39.8%) is moderately strong according to the classification system proposed by Reckase (1979), which also supported the unidimensionality of the IPS, a basic assumption as required by Rasch modeling.

### Substantive Validity

According to Wolfe and Smith (2007), substantive validity concerns whether the categories in the response scale empirically function well in agreement with the scale developer’s intention when capturing responses. Table 2 summarizes the statistics for evaluating the structure of the response scale according to the criteria established by Linacre (2002) for the RSM. As shown in Table 2, each response category has at least 118 observed frequency of responses, fulfilling the precondition of the RSM that requires a minimum of 10 observations in each category (Linacre, 2002). Meanwhile, as expected in Rasch modeling, the mean trait levels observed (average *B*_*n*_) and the step calibration parameter (*F*_*k*_) increased monotonically throughout the five response categories (the category response thresholds can also be graphically represented in trace lines where each curve shows the selection probability of a category of the item as a function of the latent trait; the item trace lines are not presented in the current report but available per request). Additionally, both the infit and outfit mean square fit indices of the response categories are less than the cut-off point of 2.00, suggesting that no unexpected response was observed. In all, these results demonstrated the adequacy and efficiency of the response scale for the IPS and that the five categories in the response scale functioned properly as initially expected the scale developer (Steel, 2002, 2010).

**TABLE 2:** The IPS response category statistics.

Category	Observed		Average *B*_*n*_	MNSQ		*F*_*k*_
	Count	*%*		Infit	Outfit	
1	460	13.6	−1.42	1.28	1.25	–
2	1,485	43.9	−0.47	0.93	0.91	−4.01
3	896	26.5	1.01	0.98	0.98	−1.35
4	420	12.5	2.39	0.95	0.97	0.92
5	118	3.5	4.12	1.07	1.03	4.63

*Count, frequency count of observed answers in the category; %, percentage; *B*_*n*_, person trait level; *F*_*k*_, step calibration; Infit, information-weighted mean square statistic; Outfit, outlier-sensitive mean square statistic; MNSQ, mean square fit indices.*

**FIGURE 1:**
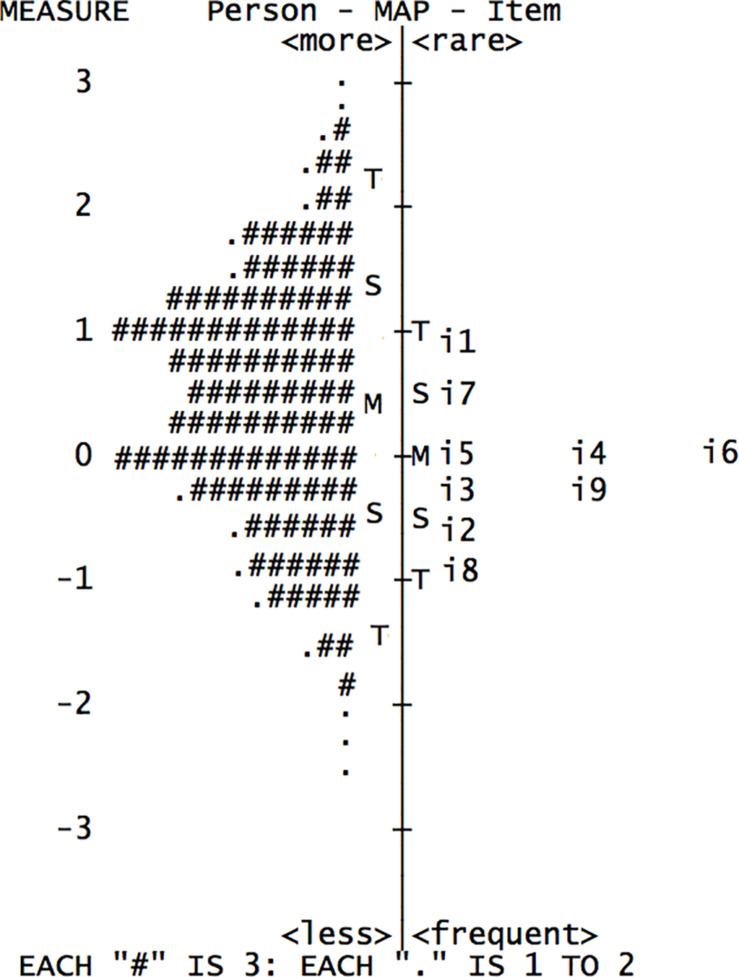
Joint person and item representations along the latent variable.

To further evaluate the strengths and weaknesses of the IPS, we consider the Wright map which allows for the evaluation of how well the scale items are distributed with regard to participants’ ability levels in the latent variable (i.e., irrational procrastination). As depicted in Figure 1, the Wright map presents the joint person and item representations along the latent variable in the identical common metric scale (i.e., logits) that expresses item difficulty from negative infinity to positive infinity (often ranging from −3 to 3 logits). The nine items of the IPS are plotted in descending order of difficulty from easiest (Item 8 on the bottom) to most difficult (Item 1 at the top) on the right side of the map and at the same time, are organized by the amount of the psychological attribute measured in the person (i.e., irrational procrastination) on the left side (the line named “Person” represents the frequency counts of participants for different points of the attribute). As shown in the graph, the estimates on both sides of the logic scale overlap substantially and the difference between the estimate means (the two “M”s on both sides of the map) is small (less than 1 logit), so that all the items can be considered appropriately directed to the students in the current sample in that the information contained in the items could allow for an accurate discrimination among people at different levels of the latent variable (Bond and Fox, 2007). In other words, the items were not too difficult or too easy for this group of respondents. However, the items appear to be mainly located in the middle range of the attribute, suggesting that the IPS provides greatest amount of information for participants with medium or medium-to-high levels of procrastination, but may not discriminate well among people with very low or high levels of procrastination. These results are in line with CTT analyses (e.g., Rozental et al., 2014; Svartdal et al., 2016; Svartdal and Steel, 2017) that showed similar findings and suggested that the IPS may have limited utility in clinical contexts where the procrastination level is expected to be high in general.

### Differential Item Functioning

To assess the validity of the IPS scores with respect to gender, DIF analysis was performed using the Mantel (1963) DIF contrast test with the Bonferroni significant level correction according to the number of comparisons (0.05/9) as recommended by Linacre (2011)^1^. DIF in Rasch modeling is established when participants with equal levels of the latent trait responded differentially to an item (Wright and Masters, 1982). The DIF analysis revealed that all IPS items functioned similarly for both gender groups in the current sample (DIF contrast was less than the cut-off point of 0.64 and Mantel–Haenszel probabilities for all items were above 0.05). All items on the IPS were thus concluded to be equitable to both male and female individuals.

## Discussion and Conclusion

Provided the methodological pitfalls in previous IPS validation studies that relied on CTT, in the present study we chose to resort to IRT using Rasch analysis in an attempt to gather complementing evidence for the psychometric properties of the IPS. Overall, the results confirmed the measurement robustness of the IPS within the Rasch modeling framework. Specifically, the results indicated good score reliability, content validity, and substantive validity of the IPS. Regarding the dimensionality, consistent with Steel’s (2010) original proposal and conclusions of the previous CTT studies (Svartdal et al., 2016; Svartdal, 2017; Kim et al., 2020), the nine items of the IPS were well fitted to the latent unidimensional structure as required in Rasch modeling which also provided support for the IPS as a unidimensional measure of irrational procrastination. The DIF analyses using a conservative criterion suggested that the functioning of the items was consistent across gender, so that one could be relatively confident that total score differences on the IPS with respect to gender may reflect true subgroup differences in the underlying construct (i.e., irrational procrastination). Although no gender difference in the IPS total scores was observed in the current study, large-scale studies have reported somewhat higher procrastination levels for men relative to women (e.g., Steel and Ferrari, 2013). Given the slightly gender-imbalance of the current sample (more females than males), the results of this study shall be used with some caution and further investigation of potential gender differences on the IPS is recommended.

Although this study focused on the application of Rasch analysis, it is worth noting that because Rasch models assume unidimensionality, they are not suitable for examining factorial structures of scales that include multiple constructs. Factor-analytic approaches, however, can accommodate multidimensional latent variables with ease, which allows for the investigation and comparison of competing single- or multi-factor models [see Gagnon et al., 2020, for a recent example of utilizing factor analysis to examine the dimensionality of the French version of the Pure Procrastination Scale (PPS; Steel, 2010)]. Given the importance of understanding the dimensionality to confirm the underlying construct of the scale (Lewis, 2017), future research may explore more powerful analyses within the structural equation modeling framework to test whether the factorial structure of the IPS would be better represented by a bifactor model (with a secondary factor associated to those three reversely scored items) than by a single-factor structure. Such evidence is particularly useful in drawing a stronger conclusion on the unidimensionality of the IPS.

Two study limitations are worth noting, particularly with regard to the sample characteristics. First, an obvious drawback pertains to the fact that the results were obtained from a college student population, and thus further research is warranted in order to try to replicate these findings in the general population. Past research (e.g., Day et al., 2000; Steel and Ferrari, 2013; Hicks and Storey, 2015) has found that the student population usually exhibits a greater level of procrastination than the general population, possibly due to the environment and age differences. Therefore, it is desirable to replicate the results from the current study with non-student samples (e.g., community sample, working adults sample). Second, although the sample used in the current study was ethnically diverse, the participants were predominantly psychology-major students and therefore, using samples consisting of more heterogeneous majors and more representative of the general body of college students would be beneficial.

To summarize, Rasch analysis was conducted to examine the psychometric properties of the IPS in the present study. Using the RSM (Andrich, 1978), we found that the IPS showed good reliability, content validity, structural validity, and substantive validity, and no DIF effects for gender. Based on the results of the current work together with previous validation studies using CTT (e.g., Svartdal et al., 2016; Svartdal, 2017; Kim et al., 2020), we thereby conclude that the IPS appears to be a compact scale with unidimensionality and the item fairness of the scale concerning gender allows for meaningful comparisons between population means in two gender groups, making it an appropriate instrument to assess individuals’ irrational procrastination (at least for the college student population) as well as a useful tool for studying gender and irrational procrastination. As such, given its relatively short length, unidimensional structure, and convenience to administer/score compared to other procrastination scales (see Steel, 2010, for a review), the IPS may be a good option for researchers who are looking for a simple, parsimonious, but effective measure of irrational procrastination to include in their studies.

## Data Availability Statement

The raw data supporting the conclusions of this article will be made available by the authors, without undue reservation.

## Ethics Statement

The studies involving human participants were reviewed and approved by the University of Houston IRB. The patients/participants provided their written informed consent to participate in this study.

## Author Contributions

Both authors contributed to the conceptualization, data collection, data analysis, and manuscript preparation.

## Conflict of Interest

The authors declare that the research was conducted in the absence of any commercial or financial relationships that could be construed as a potential conflict of interest.

## Footnotes

1 We opted to use the traditional Mantel test for simplicity and ease of interpretation. There are other ways of detecting DIF (e.g., logistic regression, IRT-based analyses) and interested readers are referred to recent studies that compared methods of assessing DIF (Sireci and Rios, 2013; Cameron et al., 2014; Shaw et al., 2020).
